# Modulation of Inflammatory Cytokine Production in Human Monocytes by cGMP and IRAK3

**DOI:** 10.3390/ijms23052552

**Published:** 2022-02-25

**Authors:** Trang H. Nguyen, Anna Axell, Ilona Turek, Bree Wright, Terri Meehan-Andrews, Helen R. Irving

**Affiliations:** 1 Department of Rural Clinical Sciences, La Trobe Rural Health School, La Trobe University, Bendigo, VIC 3552, Australia; hongtrang.nguyen@latrobe.edu.au (T.H.N.); anna-maree.axell@msd.com (A.A.); i.turek@latrobe.edu.au (I.T.); b.mellberg@latrobe.edu.au (B.W.); t.meehan-andrews@latrobe.edu.au (T.M.-A.); 2 La Trobe Institute for Molecular Science, La Trobe University, Bendigo, VIC 3552, Australia; 3 MSD Animal Health, Bendigo, VIC 3550, Australia

**Keywords:** interleukin-1 receptor-associated kinase 3 (IRAK3 or IRAK-M), cyclic guanosine monophosphate (cGMP), guanylate cyclase, nitric oxide, nuclear factor kappa-light-chain enhancer of activated B cells (NFκB), inflammatory cytokines

## Abstract

Interleukin-1 receptor-associated kinase-3 (IRAK3) is a critical checkpoint molecule of inflammatory responses in the innate immune system. The pseudokinase domain of IRAK3 contains a guanylate cyclase (GC) centre that generates small amounts of cyclic guanosine monophosphate (cGMP) associated with IRAK3 functions in inflammation. However, the mechanisms of IRAK3 actions are poorly understood. The effects of low cGMP levels on inflammation are unknown, therefore a dose–response effect of cGMP on inflammatory markers was assessed in THP-1 monocytes challenged with lipopolysaccharide (LPS). Sub-nanomolar concentrations of membrane permeable 8-Br-cGMP reduced LPS-induced NFκB activity, IL-6 and TNF-α cytokine levels. Pharmacologically upregulating cellular cGMP levels using a nitric oxide donor reduced cytokine secretion. Downregulating cellular cGMP using a soluble GC inhibitor increased cytokine levels. Knocking down IRAK3 in THP-1 cells revealed that unlike the wild type cells, 8-Br-cGMP did not suppress inflammatory responses. Complementation of IRAK3 knockdown cells with wild type IRAK3 suppressed cytokine production while complementation with an IRAK3 mutant at GC centre only partially restored this function. Together these findings indicate low levels of cGMP form a critical component in suppressing cytokine production and in mediating IRAK3 action, and this may be via a cGMP enriched nanodomain formed by IRAK3 itself.

## 1. Introduction

Inflammation is part of the host defensive reaction generated in response to infection, trauma or toxic exposure. Inflammatory responses activated by cytokine and chemokine production are decisive in eliminating pathogenic agents and repairing tissues, leading to host recovery from infection or injury, thus forming a major component of innate immunity [1]. However, hyper-inflammatory processes cause dysregulated excessive cytokine and chemokine production, known as a cytokine storm, that is linked to multi-organ dysfunction or failure frequently seen in patients with severe conditions such as sepsis or septic shock and coronavirus disease (COVID-19) [1,2].

Excessive cytokine and chemokine production is necessarily suppressed by checkpoint molecules responsible for maintaining homeostasis of inflammation [1,2]. Interleukin-1 receptor-associated kinase-3 (IRAK3 also called IRAK-M) is one checkpoint molecule, mainly expressed in immune cells such as monocytes, macrophages, dendritic cells and epithelial cells [3,4]. Similar to other members of this family (IRAK1, 2, and 4) IRAK3 is a cytoplasmic regulator of Toll-like receptor or Interleukin-1 receptor (TLRs/IL-1R) signalling pathways [3,5,6]. Members of the IRAK family have a conserved domain architecture including an N-terminal death domain, kinase (in IRAK1 and 4) or pseudokinase (in IRAK2 and 3) domain, and C-terminal domain or tumour necrosis factor receptor-associated factor 6 (TRAF6) binding domain (IRAK4 lacks this domain) [7,8,9]. Whereas other IRAKs play roles as positive regulators of TLRs/IL-1R pathways, IRAK3 acts as a negative regulator, resulting in downregulation of inflammation in the innate immune system [3,10]. The ligand engagement of TLRs or IL-1R activates the assembly of myddosome complexes composed of myeloid differentiation primary response 88 (MyD88), IRAK4, and IRAK1 or IRAK2 that interact via their death domains [8,9]. IRAK1 or 2 then dissociate from the complex and interact with TRAF6 to mediate transforming growth factor beta kinase 1 (TAK1) dependent activation of nuclear factor kappa-light-chain enhancer of activated B cells (NFκB), eventually leading to the production of pro-inflammatory cytokines such as tumour necrosis factor-α (TNF-α) and interleukin-6 (IL-6) [8,9]. IRAK3 downregulates the signal cascade by inhibiting the dissociation of IRAK-TRAF6 from the receptor–myddosome complex [3,10]. The suppressive effect of IRAK3 on inflammatory responses via NFκB activation pathway (or other pathways such as inhibiting translational control of pro-inflammatory cytokines) subtly occur at different time points depending on cell type, mode and concentration of stimuli [10,11,12,13]. IRAK3 also promotes expression of inflammatory mediators (NFκB and IL-8) upon stimulation with IL-1 or IL-1β [5,7], and induces IL-6, IL-5 and IL-13 production in response to extracellular IL-33 stimulation [12]. Therefore, the regulatory mechanism of IRAK3 function in inflammation is not fully understood.

IRAK3 contains a cryptic guanylate cyclase (GC) centre, capable of producing cyclic guanosine monophosphate (cGMP) from guanosine triphosphate, embedded within the pseudokinase domain and connected with its suppressive inflammatory function [14,15]. Since cGMP also suppresses inflammatory responses [16,17,18,19], it is possible that there is an association between cGMP and IRAK3 actions. cGMP synthesis by GCs is enhanced in response to various signals including nitric oxide (NO), peptide ligands or fluxes in intracellular calcium ions [20]. There are two well-known classes of GCs: soluble GCs and transmembrane GCs. Soluble GCs are stimulated by the binding of NO (a free radical gas synthesized by nitric oxide synthases) to the GC heme cofactor [21,22]. Transmembrane GCs are integral plasma membrane receptors containing an intracellular GC domain and activated by the extracellular atrial or A-type, brain or B-type and C-type natriuretic peptides (ANP, BNP and CNP) to generate cGMP [23]. Sophisticated interplay or integration of several messenger molecules such as cGMP or cyclic adenosine monophosphate (cAMP) with calcium ions and strict intracellular compartmentalization of these messengers is important in mediating cyclic nucleotide signalling [24,25]. Generating ordered spatial and temporal and stimulus-specific intracellular messages requires confining messengers (cGMP or cAMP) within defined cytoplasmic areas or nanoenvironment(s) involving the accurate intracellular placement of enzyme and scaffold protein components of signal cascades [24,26,27]. 

cGMP has different effects on inflammation under stimulated versus unstimulated conditions [28]. In unstimulated conditions, treatment with GC activators (e.g., NO donor, ANP) enhances expression of inflammatory mediators including NFκB activity and pro-inflammatory cytokines [28,29,30,31,32]. Whereas these cGMP modulating treatments attenuate inflammatory responses in LPS- or TNF-α-stimulated conditions [28,31,32,33,34,35,36,37,38,39,40,41,42]. Decreasing GC activity and thus cGMP formation by using GC inhibitors suppresses cGMP effects on inflammation in both unstimulated and LPS-stimulated conditions [29,30,39]. However, there were controversial results; for instance in LPS stimulated conditions cGMP treatment increases TNF-α protein levels [43] and downregulating cellular cGMP with GC inhibitors reduced LPS-induced TNF-α production [29,30]. Notably, the impact of NO on inflammation appears to be more complicated. NO is produced by NO synthases from stimulated innate immune cells and metabolised to other reactive nitrogen species that have cytocidal effects on pathogenic microbes [44]. NO also links innate and adaptive immunity via regulating T-cell activation and functions [44,45]. NO has biphasic actions as it increases NFκB and IL-6 mRNA and protein levels at lower concentrations (≤10 µM) and decreases these inflammatory markers at higher concentrations (>100 µM) [39]. 

The GC activity of IRAK3 is similar to those reported in several plant receptor kinases with cryptic GC activity [24,26]. A mutation in the IRAK3 GC centre reduces cGMP production and disables the inhibitory impact of IRAK3 on LPS-induced NFκB activity [14], indicating the importance of this GC centre in regulating IRAK3 function. In a study by Freihat et al. [14], the addition of sub-nanomolar levels of membrane permeable cGMP reversed the impairment of LPS-induced NFκB activity seen with the IRAK3 GC centre mutant. Thus, it is feasible that there is a direct interaction between cGMP and IRAK3 on inflammation. We hypothesise that low amounts of cGMP generated by IRAK3 may form a nanoenvironment surrounding IRAK3 and contribute to its actions. As there is a shortage of data on the inflammatory effects of cGMP at low (sub-nanomolar) levels, our study investigates how different levels of cGMP influence expression of inflammatory markers (NFκB activity, IL-6 and TNF-α) in monocytes where IRAK3 is endogenously expressed. Cellular cGMP levels were altered by direct addition of membrane permeable cGMP (8-Br-cGMP), or by treatment with pharmacological agents. To elucidate the role of IRAK3 and cGMP, we generated IRAK3 knockdown THP-1 monocyte cell lines and undertook complementation studies with wild type and loss-of-function IRAK3 mutants to further examine the mechanisms involved in modulating cytokine production.

## 2. Results

### 2.1. Sub-Nanomolar cGMP Suppresses Cytokine Production in LPS-Challenged Monocytes

**Figure 1:**
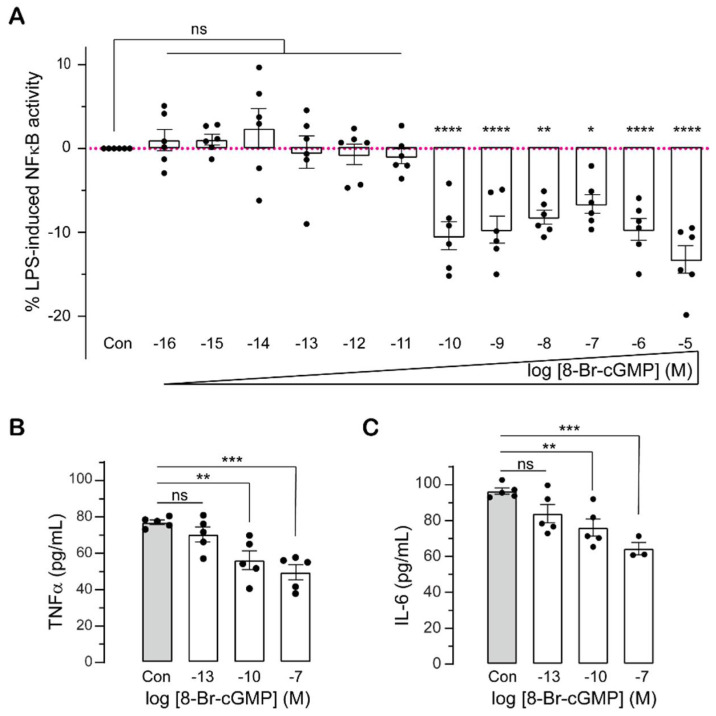
Effects of cGMP on inflammatory markers (NFκB activity, TNF-α and IL-6 production) in monocyte cell lines. (**A**) 8-Br-cGMP affects LPS-induced NFκB activity in THP-1-BLUE cells. THP-1-BLUE cells were treated with 1 µg/mL LPS and 8-Br-cGMP at concentrations from 0.1 fM to 10 µM (10^−16^ to 10^−5^ M) for 24 h, and SEAP activity correlating to NFκB activity was measured and normalised to control (Con) cells treated with LPS only (mean ± SEM, *n* = 6, One-way ANOVA followed by Dunnett’s multiple comparisons test, * *p* < 0.05, ** *p* < 0.01, *** *p* < 0.001, **** *p* < 0.0001, ns: not significant). (**B**,**C**) 8-Br-cGMP affects LPS-induced TNF-α (**B**) and IL-6 (**C**) protein production in THP-1 cells. THP-1 cells were treated with 1 µg/mL LPS and 8-Br-cGMP at 100 fM (10^−13^ M), 100 pM (10^−10^ M) and 100 nM (10^−7^ M), and 24 h post-treatment cell supernatants were collected for quantification of TNF-α and IL-6 protein levels using ELISA. Control (Con) cells were treated with LPS only (mean ± SEM, *n* = 3–5, One-way ANOVA followed by Dunnett’s multiple comparisons test, ** *p* < 0.01, *** *p* < 0.001, ns: not significant).

As the effects of low intracellular levels of cGMP on cytokine production have not been investigated, we used the membrane permeable analogue of cGMP (8-Br-cGMP) at a wide range of concentrations from sub-femtomolar to micromolar, to assess cGMP impact on inflammation by quantifying NFκB activity and cytokine production in monocytic cells. We used THP-1-BLUE cells which are derived from human THP-1 monocyte cell line and are stably integrated with an NFκB-inducible secreted embryonic alkaline phosphatase (SEAP) reporter construct (https://www.invivogen.com/seap-reporter-gene-system last accessed 30 September 2021) to study NFκB activity. LPS stimulation enhanced NFκB activity in THP-1-BLUE cells in a dose- and time-dependent manner (Supplemental Figure S1A). Stimulation with 0.01 µg/mL LPS only caused significant increases in NFκB activity at 18 h and 24 h (Supplemental Figure S1A). At LPS concentrations between 0.1 to 10 µg/mL, NFκB activity remained unchanged in the first 4 h post-treatment, then significantly increased (Supplemental Figure S1A). The highest increases in NFκB activity were observed 24 h post-treatment with 1–10 µg/mL LPS (Supplemental Figure S1A). Treatment with at least 0.1 nM (10^−10^ M) 8-Br-cGMP significantly decreased LPS-induced NFκB activity, while 8-Br-cGMP at less than 0.1 nM had no effect on NFκB activity (Figure 1A). Membrane permeable cGMP had no effect on NFκB activity in cells that have not been stimulated with LPS (Supplemental Figure S1B).

Activation and nuclear translocation of NFκB transcription factor triggers the transcription of inflammatory genes including IL-6 and TNF-α. To see if low levels of membrane permeable cGMP modulate cytokine production, we measured TNF-α and IL-6 protein secretion following LPS stimulation of THP-1 monocytes. 8-Br-cGMP at sub-nanomolar (0.1 nM) or higher (100 nM) concentrations suppressed TNF-α and IL-6 production in a dose-dependent manner (Figure 1B,C). Like NFκB activation (Figure 1A), femtomolar concentrations of 8-Br-cGMP did not affect TNF-α and IL-6 protein expression and secretion (Figure 1B,C). 

### 2.2. Pharmacologically Altering cGMP Levels Changes Inflammatory Responses

**Figure 2:**
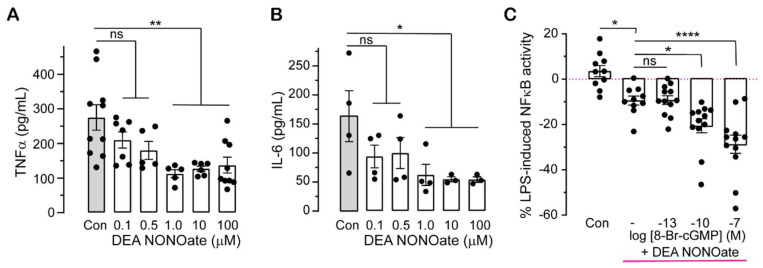
Effects of NO donor (DEA NONOate) on cytokine production and NFκB activity. (**A**) and (**B**) NO donor decreases LPS-induced TNF-α (**A**) and IL-6 (**B**) production. THP-1 cells were treated with 1 µg/mL LPS without (control; Con) or with DEA NONOate at indicated concentrations, and 24 h post-treatment cell supernatants were collected to quantify TNF-α and IL-6 protein levels using ELISA. Control cells (Con) were treated with LPS only (mean ± SEM, *n* = 3–10, One-way ANOVA followed by Dunnett’s multiple comparisons test, * *p* < 0.05, ** *p* < 0.01, ns: not significant). (**C**) 8-Br-cGMP further reduces LPS-induced NFκB activity decreased by NO donor. THP-1 BLUE cells treated with 1 μg/mL LPS only were control cells (Con). THP-1 BLUE cells treated with 1 µg/mL LPS and 1 µM DEA NONOate in the absence (indicated as “-“ in the graph) or presence of 8-Br-cGMP at 100 fM (10^−13^ M), 100 pM (10^−10^ M) and 100 nM (10^−7^ M) for 24 h; SEAP activity correlating to NFκB activation was measured and normalised to control cells (Con) (mean ± SEM, *n* = 10–12, One-way ANOVA, Dunnett’s comparison test, * *p* < 0.05, **** *p* < 0.0001, ns: not significant).

To test if changes in intracellular cGMP altered LPS-induced NFκB activity or cytokine production, we employed pharmacological modulators of soluble GCs. As NO activates cGMP generation by soluble GCs [20], we used different concentrations of the NO donor (DEA NONOate) to determine if endogenous cGMP affects inflammatory responses. TNF-α and IL-6 protein levels are significantly downregulated at micromolar concentrations of the NO donor in LPS-stimulated THP-1 cells (Figure 2A,B). To confirm whether the effect of the NO donor on inflammatory signals responds to additional cGMP, monocytes were treated with the NO donor (at 1 µM), simultaneously with 8-Br-cGMP and LPS for 18–24 h. NFκB activity reduced by NO in LPS-stimulated cells is further decreased by membrane permeable cGMP at 0.1 nM (10^−10^ M) and 100 nM (10^−7^ M) (Figure 2C).

**Figure 3:**
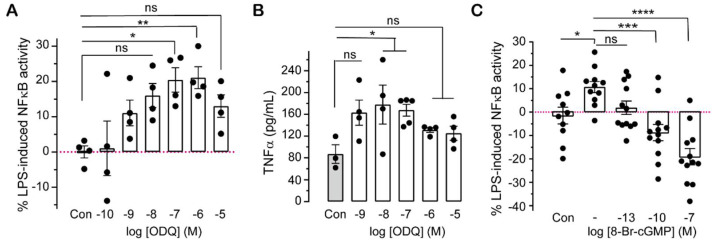
Effects of ODQ (a soluble GC inhibitor) on LPS-induced NFκB activity and TNF-α production. (**A**) ODQ stimulates LPS-induced NFκB activity. THP-1 BLUE cells were treated with 1 µg/mL LPS with or without ODQ at concentrations from 0.1 nM to 10 µM (10^−10^ to 10^−5^ M), 24 h post-treatment SEAP activity correlating to NFκB activation was measured and normalised to control cells (Con) treated with LPS only (mean ± SEM, *n* = 4, One-way ANOVA followed by Dunnett’s multiple comparisons test, * *p* < 0.05, ** *p* < 0.01, ns: not significant). (**B**) ODQ stimulates LPS-induced TNF-α production. THP-1 cells were treated with 1 µg/mL LPS with or without ODQ at concentrations from 1 nM to 10 µM (10^−9^ to 10^−5^ M), and 24 h post-treatment cell supernatants were collected to quantify TNF-α protein levels by using ELISA. Control cells (Con) were treated with LPS only (mean ± SEM, *n* = 3–5, One-way ANOVA followed by Dunnett’s multiple comparisons test, * *p* < 0.05, ns: not significant). (**C**) 8-Br-cGMP reverses the effect of ODQ on LPS-induced NFκB activity. THP-1 BLUE cells treated with 1 μg/mL LPS only were control cells (Con). THP-1 BLUE cells treated with 1 µg/mL LPS and 100 nM (10^−7^ M) ODQ in the absence (indicated as “-” in the graph) or presence of 8-Br-cGMP at 100 fM (10^−13^ M), 100 pM (10^−10^ M) and 100 nM (10^−7^ M) for 24 h; SEAP activity correlating to NFκB activation was measured and normalised to control cells (Con) (mean ± SEM, *n* = 10–12, One-way ANOVA, Dunnett’s comparison test, * *p* < 0.05, *** *p* < 0.001, **** *p* < 0.0001, ns: not significant).

To determine whether downregulation of cellular cGMP levels influences inflammation, the effect of ODQ, an inhibitor of soluble GC, on NFκB activity and cytokine level was examined. ODQ at 100 nM (10^−7^ M) and 1 µM (10^−6^ M) enhanced LPS-induced NFκB activity (Figure 3A), while 10–100 nM (10^−8^–10^−7^ M) ODQ resulted in significant increases in LPS-mediated TNF-α protein production (Figure 3B). However, the effect of ODQ at higher concentrations (1 or 10 µM) tends to be weaker, which may be due to impairment of cell viability by ODQ (approximately 15% reduction in cell viability between untreated cells and cells treated with 10 µM ODQ; Supplemental Figure S2). To check if the effect of ODQ on inflammation is cGMP dependent, cells were treated simultaneously with ODQ and 8-Br-cGMP. A concentration of ODQ of 100 nM was selected as it did not affect cell viability (Supplemental Figure S2) but significantly enhanced NFκB activity and TNF-α levels (Figure 3A,B). The influence of 100 nM ODQ on LPS-stimulated NFκB activation was reversed by the addition of at least 0.1 nM (10^−10^ M) 8-Br-cGMP (Figure 3C). 

### 2.3. Effects of cGMP on Inflammatory Markers in Wild Type and IRAK3 Knockdown/Knockout THP-1 Cells

IRAK3 inhibits NFκB activity and cytokine production [3] and also contains a cryptic GC centre that generates cGMP [14]. To elucidate the relationship between IRAK3 and cGMP on inflammatory cytokine production, we first investigated the effect of cGMP on IRAK3 protein expression in THP-1 cells. Membrane permeable cGMP over the concentration range of 1 pM–10 µM (10^−12^ to 10^−5^ M) had no effect on IRAK3 protein expression in THP-1 cells in either unstimulated or LPS-stimulated conditions (*n* = 5; Supplemental Figures S3 and S4). This finding indicates that cGMP does not induce or inhibit IRAK3 expression under the conditions used.

**Figure 4:**
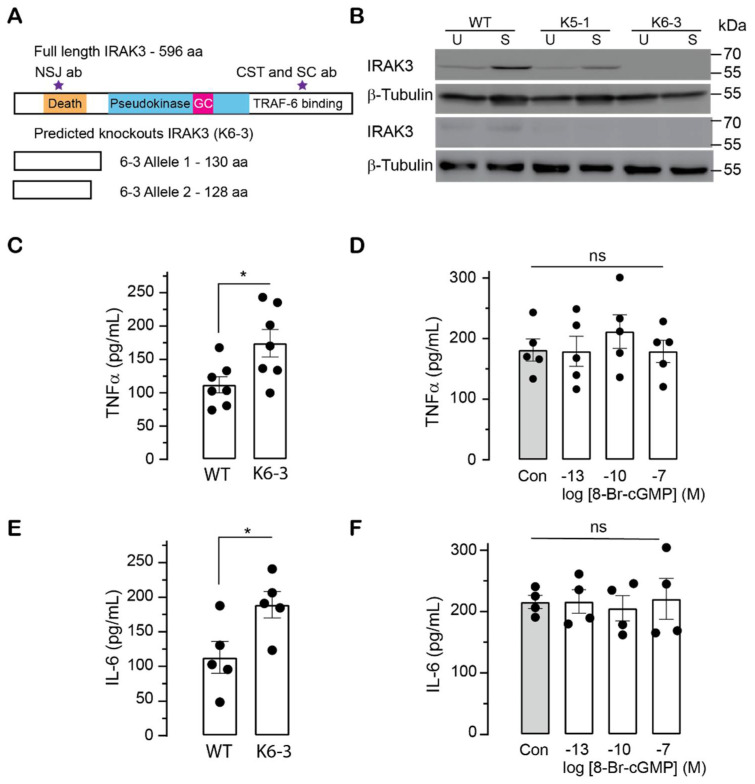
IRAK3 protein expression and cytokine production in THP-1 wild type (WT) cells versus IRAK3 knockdown/knockout THP-1 cells. (**A**) The full length IRAK3 protein containing 596 amino acids has a death domain, pseudokinase domain embedded (purple stars) with a GC centre and TRAF6 binding domain (C-terminal domain). Positions where the anti-IRAK3 antisera bind to the death domain (NSJ Bioreagent) or C-terminal domain (Cell Signalling Technology—CST) are indicated on the full length IRAK3 protein schematic. The predicted knockouts of IRAK3 in K6-3 cell line contain one allele with 128 amino acids and a second allele with 130 amino acids producing unique truncated proteins with no similarity to wild type IRAK3 with 596 amino acids. (**B**) IRAK3 protein expression in the IRAK3^+/−^ K5-1 or IRAK3^−/−^ K6-3 THP-1 cells is reduced compared to wild type IRAK3^+/+^ THP-1 cells. Cells were un-stimulated or mock PBS-treated (annotated as U in the blots), or stimulated with 1 µg/mL LPS (annotated as S in the blots) for 16 h, upon which cell lysates were collected for immunoblot experiments. IRAK3 was detected with the CST (upper panel) or NSJ (lower panel) antisera; β-tubulin was used as the loading control. Full-length immunoblots are shown in Supplemental Figure S8. (**C**,**E**) LPS-induced TNF-α and IL-6 levels were significantly increased in K6-3 THP-1 IRAK3^−/−^ cells compared to THP-1 IRAK3^+/+^ wild type cells. THP-1 IRAK3^+/+^ wild type or K6-3 THP-1 IRAK3^−/−^ cell lines were stimulated with 1 µg/mL LPS for 18 h and cell supernatants were collected to quantify TNF-α (**C**) and IL-6 (**E**) cytokine protein levels (mean ± SEM, *n* = 5–7, Unpaired *t*-test, * *p* < 0.05). (**D**,**F**) cGMP has no effect on cytokine production in K6-3 THP-1 IRAK3^−/−^ cells. K6-3 THP-1 IRAK3^−/−^ cells were treated with 1 µg/mL LPS and 8-Br-cGMP at 100 fM (10^−13^), 100 pM (10^−10^) and 100 nM (10^−7^), and 24 h post-treatment cell supernatants were collected to quantify TNF-α (**D**) and IL-6 (**F**) protein levels using ELISA. Control cells (Con) were treated with LPS only (mean ± SEM, *n* = 4–5, One-way ANOVA followed by Dunnett’s multiple comparisons test, ns: not significant).

To examine if cGMP modulates IRAK3 effects on inflammation, the IRAK3 gene in THP cells was inactivated using a CRISPR-Cas9 approach. We designed sgRNA primers targeting two sites at exon 1 of IRAK3 gene (Supplemental Figure S5) and co-transfected these with Cas9-GFP protein into THP-1 cells. Two IRAK3 knockdown monoclonal cell lines designated K5-1 and K6-3 were obtained using the limited dilution cloning method. Analysis of PCR products showed the cleavage occurred only at one of two sites in IRAK3 knockdown K5-1 and K6-3 cell lines (Supplemental Figure S6A). Sanger sequencing showed deletion of one nucleotide at one allele, and deletion of seven nucleotides at the other allele in the K6-3 line (Supplemental Figure S6B), indicating K6-3 is a heterozygous IRAK3 mutant. These nucleotide deletions in K6-3 cells cause separate frameshift mutations and early introduction of a stop codon to terminate translation, predicted to result in two different versions of truncated proteins with no similarity to IRAK3 (Figure 4A). Sanger sequencing showed a nucleotide substitution at one allele and deletion of two nucleotides at the other allele in the K5-1 line (Supplemental Figure S6B), indicating K5-1 is a heterozygous IRAK3 mutant. The nucleotide substitution in one allele of IRAK3 gene of K5-1 led to one amino acid substitution (N4T) in IRAK3 protein mutant, and the deletion of two nucleotides causes a frameshift mutation and early introduction of a stop codon, resulting in a truncated protein with no similarity to IRAK3 (Supplemental Figure S7A).

To test the level of IRAK3 protein present in the cell lines, we used three commercial IRAK3 antibodies which bind to the death domain (NSJ Bioreagent) or C-terminal domain (Santa Cruz and Cell Signaling Technology—CST) (Figure 4A). IRAK3 protein is virtually undetectable with any of the antisera in the K6-3 THP-1 IRAK3 heterozygous knockout (IRAK3^−/−^) cells compared to wild type cells (Figure 4B and Supplemental Figure S8). However, light bands of IRAK3 wild type protein occur in K5-1 THP-1 IRAK3 heterozygous knockdown (IRAK3^+/−^) cells (Figure 4B and Supplemental Figure S8), as the CRISPR/Cas9-mediated mutations generated a truncated protein with only 44 amino acids and a full length IRAK3-mutated protein with one amino acid substitution (Supplemental Figures S6B and S7A). Thus, the K5-1 IRAK3^+/−^ cell line is not a full knockout with partial expression of IRAK3-mutated protein containing the substituted amino acid (N4T) that is not predicted to affect IRAK3 function.

Treatment with LPS markedly stimulated production of TNF-α and IL-6 cytokines in K5-1 IRAK3^+/−^ or K6-3 IRAK3^−/−^ monocytes compared with wild type monocytes (Figure 4C,E and Supplemental Figure S7B,D), confirming the suppressive effects of IRAK3 on inflammatory responses to endotoxins reported previously [3,4,10,11,46]. To determine if cGMP could reduce LPS-induced cytokine production, the K6-3 IRAK3^−/−^ cells were simultaneously exposed to LPS and 8-Br-cGMP, and no change in TNF-α and IL-6 protein levels were seen (Figure 4D,F). A similar response is observed for the heterozygous K5-1 THP-1 IRAK3^+/−^ mutants (Supplemental Figure S7C,E). The lack of response markedly differs from LPS-stimulated wild type cell lines where TNF-α and IL-6 levels are significantly suppressed by the same doses of cGMP (0.1 nM and 100 nM) (Figure 1B,C). This finding indicates that there is a relationship between the effects of cGMP and IRAK3 on regulating immune responses in monocytic cell lines.

### 2.4. Effect of Mutations in IRAK3 on Cytokine Level

**Figure 5:**
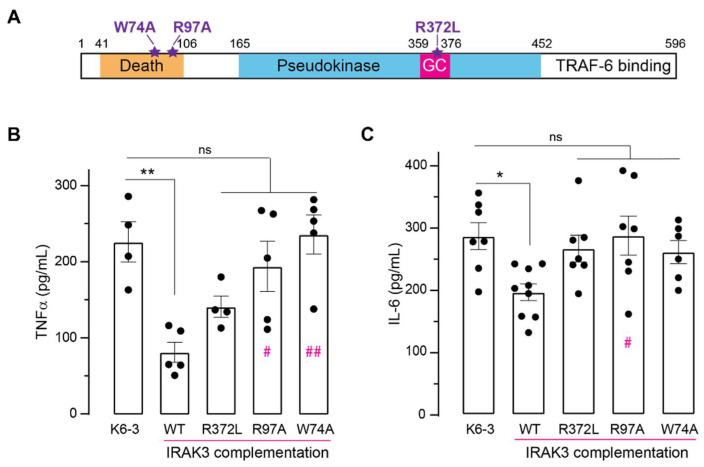
Complementation of K6-3 THP-1 IRAK3^−/−^ cells with wild type and mutant IRAK3 proteins. (**A**) Schematic of IRAK3 domain architecture indicating point mutations (purple stars) introduced in the death domain (W74A, R97A) and in the GC centre (R372L). (**B**,**C**) TNF-α and IL-6 protein levels in K6-3 THP-1 IRAK3^−/−^cells complemented with IRAK3 wild type (WT) or specific IRAK3 mutants. K6-3 THP-1 IRAK3^−/−^ cells were transfected with vectors carrying wild type or mutated IRAK3 gene; untransfected K6-3 THP-1 IRAK3^−/−^ cells were used as the negative control. After transfection, the cells were treated with 1 µg/mL LPS for 24 h, and cell supernatants were collected for quantification of TNF-α and IL-6 protein levels using ELISA (mean ± SEM, *n* = 4–9, One-way ANOVA followed by Tukey’s multiple comparisons test; * *p* < 0.05, ** *p* < 0.01, ns not significant, pink hash mark (#) in columns indicates the statistically significant difference between WT and IRAK3 mutants, ^#^ *p* < 0.05, ^##^ *p* < 0.01).

To study how different domains of IRAK3 influence its effects on production of inflammatory cytokines, the knockdown cell lines were complemented with wild type and mutant versions of IRAK3 protein (Figure 5A). K6-3 IRAK3^−/−^ or K5-1 IRAK3^+/−^ cells transfected with IRAK3 wild type exhibited significant decreases in LPS-induced IL-6 and TNF-α protein levels compared to the level of untransfected cells (Figure 5B,C and Supplemental Figure S9), further confirming IRAK3 inhibits inflammatory responses [3,10,11]. The IRAK3 R372L mutant decreases GC activity and fails to reduce LPS-induced NFκB activity [14]. Transfection of K6-3 IRAK3^−/−^ or K5-1 IRAK3^+/−^ cell lines with the R372L IRAK3 mutant did not significantly suppress LPS-induced IL-6 and TNF-α protein level compared to the level of untransfected cells (Figure 5B,C and Supplemental Figure S9). The death domain is important in the interaction of IRAK3 with other members of the IRAK family and MyD88 [7]. Hence, we sought to determine if the complementation of the K6-3 IRAK3^−/−^ or K5-1 IRAK3^+/−^ cell lines with IRAK3 containing mutations in the death domain (R97A and W74A) affected LPS-induced TNF-α and IL-6 production. Similar to the R372L mutant, the W74A and R97A mutants failed to decrease LPS-induced IL-6 and TNF-α protein expression (Figure 5B,C and Supplemental Figure S9). Thus, these mutations impair the ability of IRAK3 to inhibit expression of inflammatory cytokines, indicating both the GC centre and death domain are critical for IRAK3 functions in inflammation.

## 3. Discussion

**Figure 6:**
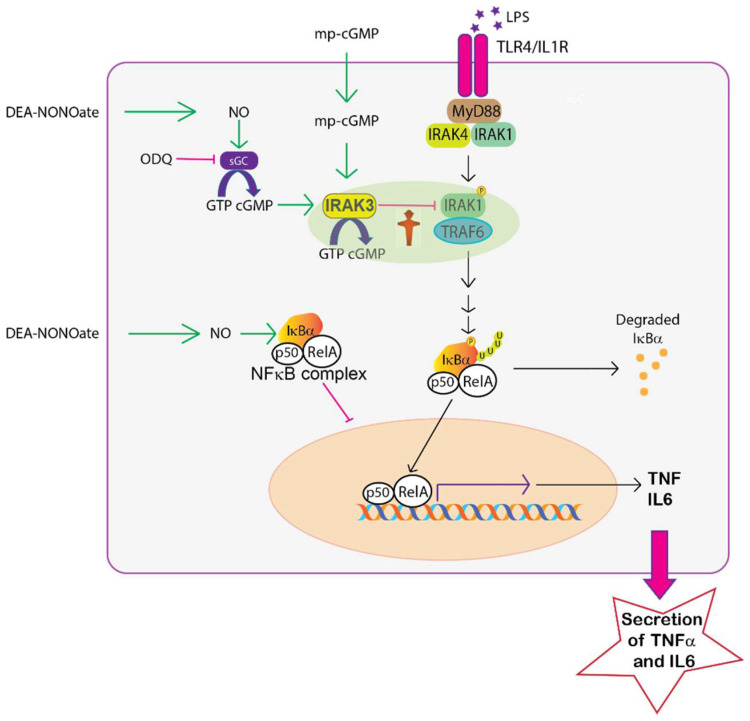
Schematic of a proposed mechanism for the interaction of IRAK3 and cGMP to modulate inflammatory cytokine production in TLR4/IL1R signalling pathway. Activation of TLR4/IL1R by endotoxins such as LPS (indicated by stars) leads to the formation of myddosome complexes comprising MyD88, IRAK4 and IRAK1/IRAK2, then activates TRAF6, that transduces the signal to degrade IκBα (an NFκB inhibitor) and trigger nuclear translocation of the transcription factor NFκB containing p50 and RelA subunits. NFκB induces the transcription of inflammatory genes (TNF-α and IL-6), consequently leading to cytokine production. IRAK3 generates cGMP to create a nanoenvironment surrounding its protein interactome and inhibits the association of IRAK1 or 2 with TRAF6, thus suppressing the downstream cascade. Sub-nanomolar levels of cellular cGMP directly added via membrane permeable cGMP (mp-cGMP) or pharmacologically modulated using DEA NONOate (NO donor) or ODQ (a soluble GC inhibitor), lead to changes in NFκB activity and cytokine production. NO released from NO donor also stabilizes IκBα by inhibiting its degradation from NFκB, causing reduced NFκB activity [49]. Figure drawn in Adobe Illustrator, red stop ampel man from Wiki commons image downloaded 3 June 2020.

There is still a great need to understand the mechanisms to fine tune inflammatory immune responses to add to the repertoire of existing anti-inflammatory drugs for treatments of inflammatory diseases such as allergy, asthma and ulcerative colitis. It is noteworthy that glucocorticoids which are a highly effective mainstay treatment for inflammatory conditions upregulate expression and activity of IRAK3 [4], thus demonstrating that modulating IRAK3 activity is a viable therapeutic target. In addition, cGMP regulates many biological processes [20], and GC inhibitors and activators (e.g., Nesiritide, Sildenafil, Riociguat) have been developed as pharmaceutical therapies for pulmonary hypertension, cardiovascular diseases, angina pectoris or erectile dysfunction [47,48]. Sildenafil, a phosphodiesterase inhibitor used for the treatment of blood hypertension and erectile dysfunction, also influences the proliferation of regulatory T cells and pro-inflammatory cytokines [18]. Those data underlie the immunomodulatory effects and potential use of GC inhibitors and activators as therapeutic agents in immune diseases but clinical data in the human immune system are limited. Hence, it is necessary to gain further insights into mechanism(s) of cGMP regulation in human immune responses. Here, we explored the association of IRAK3—an immune checkpoint molecule—with cGMP, as IRAK3 has GC activity and is predicted to generate a cGMP nanoenvironment surrounding itself and interacting proteins (Figure 6) [14,26]. We show that low levels of cGMP can reduce monocytic TNF-α and IL-6 output and that this depends upon the abundance of IRAK3. The results highlight the need for IRAK3 protein to contain an active death domain for binding to protein complexes and to a lesser extent to contain an active GC centre. Moreover, the results point to the possibility of repurposing existing drugs (at low doses) that modify cGMP levels to facilitate IRAK3 action in suppressing cytokine production.

The effects of cGMP across a large concentration range from femtomolar to micromolar were examined to fill a gap left by most prior studies that only investigated higher concentrations of cGMP in the micromolar to millimolar range [28,29,31,32,34,39,40,41,42,43]. cGMP at sub-nanomolar (0.1 nM) levels is surprisingly effective at downregulating NFκB activity, and pro-inflammatory cytokine (TNF-α and IL-6) levels induced by LPS challenge in monocytes (Figure 1). We also report that higher cGMP concentrations are effective and these findings agree with previous studies reporting the inhibitory effect of higher cGMP concentrations (1 µM–1 mM) on inflammation in LPS-stimulated cells [28,34,42,50]. cGMP has no effect on NFκB activity in unstimulated human monocytes (Supplemental Figure S1B), and this is consistent with previous results [41,51], although other studies showed that cGMP at 10 µM increased LPS-unstimulated NFκB activity and cytokine expression [28,32,39]. The reasons for these differences are unclear but may be attributed to the measured outcomes, NFκB—DNA binding activity [28,39,49] versus the NFκB SEAP reporter assay used in this study. 

Pharmacologically increasing cellular cGMP levels by treatment with the NO donor (DEA NONOate, a soluble GC stimulator) also decreased LPS-induced NFκB activity and pro-inflammatory cytokines (Figure 2A,B). Membrane permeable cGMP at 0.1 nM and 100 nM further decreased NFκB activity lowered by NO in LPS-stimulated cells (Figure 2C). This agrees with previous reports showing NO or other GC activators such as ANP inhibit NFκB activation and pro-inflammatory cytokine expression [28,32,52]. However, in unstimulated conditions, NO has biphasic effects, including stimulatory action on inflammatory responses at lower (≤10 µM) and inhibitory actions at higher (>100 µM) concentrations [39] in a cGMP-dependent manner [32,33]. NO is synthesised along with L-citrulline from L-arginine in a reaction catalysed by NO synthase [53]. Thus, diminished bioavailability of L-citrulline and L-arginine can indicate a reduction in NO production, in turn resulting in increased levels of inflammatory cytokines. Notably, IRAK3 is highly expressed in severe asthma and COVID-19 cohorts, where levels of L-citrulline and L-arginine are decreased in sera of COVID-19 and asthma patients [53,54]. These changes may contribute to the cytokine storm occurring in COVID-19 patients and is consistent with our data showing increasing NO donor reduces LPS-induced levels of TNF-α and NFκB activity (Figure 2A,B). Although IRAK3 gene expression is raised in severe acute respiratory syndrome coronavirus 2 (SARS-CoV-2)-infected human bronchial epithelial cells [53], treatment with SARS-CoV-2 spike protein downregulated IRAK3 expression in THP1-differentiated macrophages, human peripheral blood mononuclear cells and monocytes [55]. These findings indicate that the SARS-CoV-2 infection causes a dramatic production of inflammatory cytokines by decreasing IRAK3 expression and level of NO. NO downregulates NFκB activity via differential actions that partly involve IRAK3/cGMP pathway(s) (Figure 6). NO enhances the expression of IRAK3 which in turn diminishes molecular interactions of IRAK1 or 2 with TRAF6, and downstream cascades [56] (Figure 6). Additionally, the induction and stabilization of the NFκB inhibitor, alpha (IκBα) is mediated by NO, thereby attenuating NFκB activity [49] (Figure 6). NO also suppresses the activity of caspase-1 enzyme which mediates processing of premature IL-1β and IL-18 cytokines, causing decreased IL-1β and IL-18 production [57,58]. Therefore, there is a correlation between IRAK3, cGMP and NO effects to downregulate inflammatory cytokine production.

The potential for altering cGMP levels to influence systemic inflammatory responses has been raised previously. In a polymicrobial sepsis-induced mouse model where exogeneous cGMP was administered to increase cGMP bioavailability, plasma levels of IL-6 and TNF-α are substantially reduced, and ODQ (an inhibitor of soluble GCs) showed the opposite effect [16]. Our study confirmed that pharmacologically decreasing cGMP levels by ODQ promotes LPS-induced NFκB activity and TNF-α expression (Figure 3A,B). The effect of ODQ on LPS-induced NFκB activity was reversed by simultaneously adding membrane permeable cGMP (Figure 3C), confirming the inhibitory effect of cGMP signalling on inflammation. However, there are contradictory findings about the influence of cGMP or soluble GC inhibitors on inflammation [29,30,43]. Such examples are: zaprinast (a selective inhibitor of cGMP-specific phosphodiesterases) enhanced LPS-stimulated secretion of TNF-α and IL-1β in rat microglial cells [43]; soluble GC inhibitors such as methylene blue suppressed LPS-induced secretion of inflammatory cytokines in human pulmonary macrophages [29,30]; and ODQ at 50 µM inhibited LPS-induced production of TNF-α and IL-1β in rat microglial cells [43]. We found that ODQ at 0.1–1 µM raised LPS-induced NFκB activity (Figure 3A), at 10–100 nM ODQ increased LPS-induced TNF-α level (Figure 3B). However, at higher concentrations (10 µM), ODQ decreased the viability of THP-1 human monocytes (Supplemental Figure S2), that can lead to lower expression of inflammatory markers potentially explaining the decreased effect of 10 µM ODQ (Figure 3A,B). cGMP regulates cellular protein turnover, proteasome activity enhancing the elimination of misfolded or mutated proteins [59]. Hence, treatment with higher ODQ concentration can cause impaired proteosome function or defects in protein homeostasis and directly affects cell viability. Put together, soluble GC inhibitors may influence cytokine production in concentration dependent ways that are influenced by the cell types and effects on cell viability and mechanisms of action. 

A GC centre encapsulated in the pseudokinase domain of IRAK3 is responsible for its cGMP generating activity [14,15]. The R372L mutation in the GC centre predicted to affect the affinity of GTP to IRAK3 protein decreases in vitro cGMP production by IRAK3 protein and importantly, impairs the suppressive functions of IRAK3 on LPS-induced NFκB activation [14]. The inhibitory action of IRAK3 R372L mutant on NFκB activity is recovered by supplementing cells with sub-nanomolar membrane permeable cGMP and not further improved when using higher amounts of cGMP [14]. Such low levels of cGMP correlate with the relatively low GC activity of IRAK3 (at least in vitro) and it has been suggested that IRAK3 may generate a cGMP-enriched nanodomain surrounding itself and interacting proteins [26]. Sub-nanomolar cGMP levels were confirmed to suppress inflammatory markers in LPS stimulated cells, but have no effect in the IRAK3 knockdown/knockout cells, suggesting cGMP-mediated inhibition of inflammatory cytokine production requires the normal degree of expression of wild type IRAK3 proteins in monocytes (Figure 1 and Figure 4, and Supplemental Figure S7C,E). It is possible that immunosuppressive signal is amplified in the presence of cGMP via IRAK3 acting as a checkpoint molecule upon LPS stimulation. Membrane permeable 8-Br-cGMP (0.1 nM–10 µM) reduces LPS-induced NFĸB activity in a dose-dependent manner in human embryonic kidney (HEK BLUE) cells not expressing IRAK3 [14], whereas cGMP effect is dose-independent in human monocytic THP-1 cells that natively express IRAK3 (Figure 1A). Collectively, these data imply that IRAK3 synergically acts within a nanoenvironment of cGMP to modulate inflammation.

Although IRAK3 protein expression is unaffected by cGMP treatment (Supplemental Figures S3 and S4), cGMP can modulate IRAK3 actions without changing its expression level. As IRAK3 generates cGMP, it may create a localised nanoenvironment that can inhibit inflammation or alternatively mediate IRAK3 enzymatic activity or interaction with other IRAKs or MyD88 to control the TLRs/IL-1R pathway. Complementation with IRAK3 wild type in the knockdown cells causes decreases in TNF-α and IL-6 production as expected, showing the knockdowns are effective (Figure 5 and Supplemental Figure S9). LPS-induced TNF-α and IL-6 protein levels are not significantly altered by complementation with any of the IRAK3 mutants tested (Figure 5 and Supplemental Figure S9), implicating these residues in the death domain or GC centre in enabling IRAK3 actions. The IRAK3 R372L mutant is less successful in complementing the knockdown cells than wild type protein, and not significantly different at inducing cytokine production from the control untransfected cells (Figure 5 and Supplemental Figure S9). However, the IRAK3 R372L mutant was not statistically different from the complementation with the wild type (Figure 5 and Supplemental Figure S9). One reason may be that enough basal cGMP is present in monocytes for the mutant IRAK3 to function and this mutant partially responds to additional cGMP as shown previously [14]. The other two mutated versions of IRAK3 (R97A and W74) contain mutations introduced in the death domain that are required for IRAK3 to bind to other proteins, including other IRAKs and MyD88, thus IRAK3 action on inflammation is inhibited [7,10]. Correspondingly, stably expressing either two death domain mutants in wild type THP-1 cells also raised IL-6 or TNF-α protein levels compared to IRAK3 wild type—transduced cells [7]. Complementation with either W74A or R97A mutants confirmed the importance of IRAK3 interactions via the death domain to downregulate IL-6 and TNF-α production (Figure 5 and Supplemental Figure S9). Despite not directly engaging IRAK3 interaction with myddosome complexes involved in NFκB activation cascade, GC centre still influenced IRAK3 effects on expression of inflammatory cytokines, possibly via a cGMP nanoenvironment generated by GC centre itself.

The second messenger cGMP formed by soluble GCs, that are activated by NO or deactivated by soluble GC inhibitors such as ODQ, downregulates inflammatory responses to endotoxin stimulation via IRAK3 molecules (Figure 6). Additionally, IRAK3 produces a low amount of cGMP potentially involved in regulating its functions (Figure 6). Together this suggests that the inflammatory responses can be modulated by regulating cellular cGMP levels. We show how modifying cellular cGMP levels using different approaches may contribute to the localised cGMP nanoenvironment generated by IRAK3 and thus modulating the degree of inflammation. Our results hint at the possibility of facilitating IRAK3 action by repurposing existing drugs (at low doses) that modify cGMP levels to complement other treatments such as glucocorticoids that are associated with many side effects. Our study confirms the effectiveness of very low changes in cellular cGMP levels in modulating TNF-α and IL-6 cytokine production and NFκB activity using an in vitro cell model. Further research should be performed using both in vitro and in vivo models using low therapeutic doses of drug classes such as soluble GC activators or stimulators or phosphodiesterase inhibitors [47] to manipulate cellular cGMP levels to modulate cytokine production or other inflammatory markers such as NFκB activity as these drugs may form valuable adjuncts for treating chronic inflammatory diseases. 

## 4. Materials and Methods

### 4.1. Cell Culture and Treatments

Human monocyte (THP-1) cells (American Type Culture Collection ATCC TIB-202) and THP-1-BLUE (InvivoGen, San Diego, CA, USA) are non-adherent suspension cells cultured in Roswell Park Memorial Institute (RPMI) 1640 medium (Gibco, Thermo Fisher Scientific, Melbourne, Australia) with 10% (*v*/*v*) Fetal Bovine Serum (FBS; Gibco) in 5% CO_2_ at 37 °C in a tissue culture incubator. All cells were split for 3 passages once taken from frozen stock before testing for mycoplasma using the PCR Mycoplasma Detection Kit (TOKU-E, Bellingham, WA, USA) and confirmed to be negative for mycoplasma before use. Cells were grown to 70–80% confluency before they were passaged by removing approximately 80% volume of cell medium solution in the flask and back-filled with the same volume of fresh medium and were only used up to a maximum passage number of 30. For THP-1-BLUE cells, 10 μg/mL Blasticidin S hydrochloride (Merck, Melbourne, Australia) was added every alternative passage to maintain selection pressure for NFκB-inducible secreted embryonic alkaline phosphatase (SEAP) reporter genes. For SEAP assay and ELISA experiments, cells were treated with 8-bromoguanosine 3′,5′-cyclic monophosphate sodium salt (8-Br-cGMP; Merck), or 1H-[1,2,4]oxadiazolo[4,3-a]quinoxalin-1-one (ODQ; Merck), or diethylamine NONOate sodium salt hydrate (DEA NONOate; Merck), and/or lipopolysaccharide (LPS, *Escherichia coli* 055:B5; Merck) at the indicated concentrations for 18–24 h; the negative control cells were mock-treated with phosphate-buffered saline (PBS). The stock solutions of LPS, 8-Br-cGMP and DEA NONOate were made up in PBS, and ODQ was resuspended in ethanol. These stock solutions were then diluted in PBS to make working solutions.

### 4.2. SEAP Assay

THP-1-BLUE cells were split at approximately 2 × 10^4^ cells in 200 µL medium per well in Corning Costar cell culture clear flat bottom 96-well plates and treated with 1 µg/mL LPS with the addition of 0.1 fM–10 µM 8-Br-cGMP and/or 0.1 nM–20 µM ODQ and/or 0.1 µM–100 µM DEA NONOate. Negative control THP-1-BLUE cells were treated with 1× PBS and positive control cells were treated with 1µg/mL LPS. Cells were grown for 18–24 h at 37 °C in RPMI 1640 medium supplemented with 10% FBS in a 5% CO_2_ incubator. Aliquots (30 µL) of supernatant from control or stimulated cells were transferred in duplicate to clear flat bottom 96-well plates and 180 μL of QUANTI Blue solution (InvivoGen) was added as described in the THP1-Blue NFκB Cells protocol (https://www.invivogen.com/sites/default/files/invivogen/products/files/thp1_blue_nf_kb_tds.pdf, last accessed 12 August 2021). Plates were then incubated at 37 °C for 1–2 h. SEAP levels were measured spectrophotometrically at 660 nm using the CLARIOstar^Plus^ (BMG Labtech, Ortenberg, Germany).

### 4.3. ELISA

Wild type and IRAK3 knockdown THP-1 cells were cultured at approximately 1 × 10^5^ to 2 × 10^5^ cells in 500 μL medium per well in Corning Costar cell culture clear flat bottom 24-well plates. After cell treatments with 1 µg/mL LPS and the addition of 100 fM–100 nM 8-Br-cGMP or 0.1 nM–10 µM ODQ or 0.1–100 µM DEA NONOate (control cells treated with LPS only), 100 µL of supernatant aliquots from cell cultures were collected and measured for the level of human cytokines (TNF-α and IL-6) using BD OptEIA IL-6 and OptEIA TNF-α ELISA kits (BD Biosciences, San Diego, CA, USA) according to the manufacturer’s instructions.

### 4.4. Immunoblots

THP-1 cells were lysed in protein lysis solution containing 10 mM Tris-HCl (pH 7.5), 150 mM NaCl, 0.5 mM EDTA, 0.5% (*v*/*v*) NP-40 (Merck), 1 mM phenylmethylsulfonyl fluoride (Merck) with freshly added 1X cOmplete EDTA-free protease inhibitor cocktail solution (Merck). Whole cell lysates were then separated on 10–15% SDS-PAGE gels and transferred to Hybond ECL nitrocellulose membranes (Merck). The membranes were blocked with 5% (*w*/*v*) non-fat dry milk in Tris-buffered saline (TBS) containing 0.1% (*v*/*v*) Tween 20 (TBST) for 1–2 h. The membranes were then incubated with diluted primary antibody in 5% (*w*/*w*) non-fat dry milk—TBST at 4 °C overnight. The primary antibodies and dilutions are listed in Supplemental Table S1. After washing three times for 5–10 min with TBST, the membranes were incubated with the corresponding secondary antibody as listed in Supplemental Table S1, at room temperature for 2 h, followed by three 10 min washes with TBST. The chemiluminescent signal was developed on membranes using Amersham ECL Select Western Blotting Detection Reagent (Merck) to detect the respective proteins, and images were obtained with G:Box ChemiXL1.4 (Syngene, Bangalore, India). Densitometric analyses were performed using Fiji software [60].

### 4.5. Generating IRAK3 Gene Knockdown THP-1 Cells

Two knockdown cell lines were developed using the CRISPR (Clustered Regularly Interspaced Short Palindromic Repeats)-Cas9 cleavage system. Based on human IRAK3 sequence (NG_021194.1) from the NCBI database, single guide RNA (sgRNA) sequences were designed to target IRAK3 gene using the Genetic Perturbation Platform (GPP) sgRNA Designer Software (Broad Institute), known as sgRNA1 and sgRNA2 (Supplemental Table S2). An equimolar ratio of sgRNA1, sgRNA2 (Thermo Fisher Scientific) and Cas9-GFP protein (Merck) were transfected into THP-1 cells using the Neon Transfection System (Thermo Fisher Scientific) according to the manufacturer’s instructions (transfection conditions: 1700 V/20 ms/1 pulse). Cell transfection was confirmed by observing GFP presence using an Olympus CKCx5 fluorescence microscope and Countess II Automated cell counter (Thermo Fisher Scientific). Limited dilution cloning was conducted on transfected cells grown in RPMI 1640 medium supplemented with 10% (*v*/*v*) FBS in 96-well plates to establish single-cell-derived colonies. When the cells in each well reached confluency, they were sub-cultured up to T75 flasks, cells were collected, and genomic DNA was extracted using the ISOLATE II Genomic DNA kit (Bioline, Memphis, TN, USA) following the manufacturer’s protocol. Polymerase chain reaction (PCR) was conducted to confirm CRISPR/Cas9 cleavage, using two primer sets (primer set 1: 41644-IRAK3_Fwd1 and 41820-IRAK3_Rev bind to sequences flanking the target sites of two sgRNAs; and primer set 2: 41819-IRAK3_Fwd2 binds to a sequence between the target sites of two sgRNAs and 41820-IRAK3_Rev binds to a sequence flanking the target sites of two sgRNAs) given in Supplemental Table S3, with PCR conditions given in Supplemental Table S4.

The PCR fragments were amplified using the genomic DNA of THP-1 wild type and CRISPR/Cas9-generated cell lines (K5-1 and K6-3) as templates and two primers 41644-IRAK3_Fwd3 and 41645-IRAK3_Rev (Supplemental Table S3) that bind to sequences flanking the target sites of two sgRNAs, using Q5 High-Fidelity DNA Polymerase (New England BioLabs, Ipswich, ME, USA) (initial denaturation at 98 °C for 30 s, followed by 20 cycles of 98 °C denaturation for 10 s, 65 °C annealing for 30 s and an extension at 70 °C for 2 min; final extension at 72 °C for 10 min). PCR products were cloned into the vector, pCR-4Blunt-TOPO of Zero Blunt TOPO PCR Cloning Kit (Thermo Fisher Scientific) following the manufacturer’s protocol. The clones were transformed into One Shot TOP10 competent cells (Thermo Fisher Scientific) and grown in kanamycin (50 μg/mL)-containing media for selection. Plasmids from 6 to 10 clones were purified using PureLink Quick Plasmid Miniprep Kit (Thermo Fisher Scientific) and sequenced with T3 primer at Micromon Genomics (Monash University). Whole cell protein lysates were subjected to immunoblotting using anti-IRAK3 antibodies and anti-β-tubulin antibody for loading control as described in Western Blot section.

### 4.6. Transfection of Wild Type and Mutant IRAK3 Constructs to IRAK3 Knockdown Cells

The IRAK3 wild type clone and IRAK3 mutant R372L clone were generated as described [14]. The IRAK3 mutant W74A and R97A clones were generated by site-directed mutagenesis of the IRAK3 wild type construct using specific primers (0.5 μM) (Supplemental Table S5) and Phusion High-Fidelity DNA Polymerase (New England BioLabs). The IRAK3 mutant clones were transformed to competent cells, and plasmids of these clones were purified as described [14]. All the constructs were sequenced (Micromon Monash University) and those with correct in frame insertions were kept and independently recombined into the Gateway expression vector pcDNA 6.2/C-EmGFP-DEST for mammalian cell expression (Thermo Scientific, Waltham, MD, USA) using the Gateway LR Clonase II Enzyme mix [14].

IRAK3 gene knockdown THP-1 cells grown in 6-well plates were transiently transfected with IRAK3 wild type or R372L, R97A, W74A mutant constructs in pcDNA 6.2/C-EmGFP-DEST vector [7,14], using the Lipofectamine 3000 Reagent (Thermo Fisher Scientific) following the manufacturer’s protocol. After transfection, cells were treated with 1 µg/mL LPS for 24 h, supernatants from cell cultures were collected and measured to detect the level of human cytokines (TNF-α and IL-6) present using ELISA as described above. Transfection efficiency was first checked using the Countess II Automated cell counter (Thermo Fisher Scientific), and then by measuring fluorescence intensity (excitation: 487 nm, emission: 510 nm) using the CLARIOstar^Plus^ plate reader. The total protein concentration of the transfected cells was quantified by method of [61]. The quantified cytokine level of control (untransfected or transfected with empty vector) and IRAK3 wild type or mutant—transfected cells were normalised based on fluorescence intensity and total protein concentration of these cells.

### 4.7. Statistical Analysis

Data from at least three biological replicates were analysed using GraphPad Prism 9 software (GraphPad Software, San Diego, CA, USA) by *t*-test or one-way ANOVA followed by Dunnett’s multiple comparison or Tukey’s multiple comparison post hoc tests where *p* < 0.05 was considered significant.

## Data Availability

All data are reported in the manuscript and in the Supplementary Material.

## Supplementary Materials

The supporting information can be downloaded at: https://www.mdpi.com/article/10.3390/ijms23052552/s1. References [4,14,62,63] are cited in the supplementary materials.
