# Supplementary material for: Modulation of Inflammatory Cytokine Production in Human Monocytes by cGMP and IRAK3
Source: Int J Mol Sci. 2022 Feb 25;23(5):2552. doi: 10.3390/ijms23052552 (PMC8909980; doi:10.3390/ijms23052552)
Supplement: Supplementary file 1 [file ijms-23-02552-s001.zip › ijms-1604304-supplementary.pdf]

**Supplemental document**

# Modulation of inflammatory cytokine production in human monocytes by cGMP and IRAK3

Trang H Nguyen, Anna Axell, Ilona Turek, Bree Wright, Terri Meehan-Andrews, Helen R Irving.

The following pages include:

**Supplemental Tables:**

Supplemental Table S1. List of antibodies and dilutions.

Supplemental Table S2. Sequences and positions of two single guide RNAs (sgRNAs) used in CRISPR/Cas9 system.

Supplemental Table S3. Primers used to develop and check the knockdown of IRAK3 in THP-1 cells.

Supplemental Table S4. PCR conditions.

Supplementary Table S5. Primers used in mutagenesis of IRAK3 constructs for complementation study.

**Supplemental Figures:**

Supplemental Figure S1. NFκB activity in THP1 BLUE cells after treatment with LPS or 8-Br-cGMP.

Supplemental Figure S2. THP-1 cell viability after ODQ treatment.

Supplemental Figure S3. IRAK3 protein expression in THP-1 cells after treatment with 8-Br-cGMP with or without LPS.

Supplemental Figure S4**.** Full length immunoblots of THP-1 cells after treatment with 8-Br-cGMP with or without LPS.

Supplemental Figure S5. CRISPR/Cas9 system used to knockdown IRAK3 protein.

Supplemental Figure S6. Screening and analysis of the knockdown of IRAK3 gene.

Supplemental Figure S7. Effect of cGMP on inflammatory responses in CRISPR mediated-IRAK3 knockdown cell line K5-1.

Supplemental Figure S8. Immunoblots of THP-1 wild type cells and CRISPR-mediated IRAK3 knockdown cell lines (K5-1 and K6-3).

Supplemental Figure S9. Effect of mutations in guanylate cyclase centre (R372L) and death domain (W74A, R97A) of IRAK3 on cytokine production.

**References for supplemental material:**

**Supplemental Table S1**. List of antibodies and dilutions.

| Name | Catalogue number | Company | Dilution | References |
|---|---|---|---|---|
| IRAK-M rabbit polyclonal antibody | 4369 | Cell Signalling Technology | 1:1000 | (1) |
| IRAK-M mouse monoclonal antibody | sc-100389 | Santa Cruz | 1:500 | (2) |
| IRAK-M rabbit polyclonal antibody | F50786 | NSJ Bioreagents | 1:500 | |
| β-tubulin rabbit polyclonal antibody | ab6046 | Abcam | 1:2000 | (3) |
| Anti-rabbit IgG (whole molecule) – peroxidase antibody | A0545 | Merck | 1:3000 - 1:6000 | |
| Anti-Mouse IgG (whole molecule)–peroxidase antibody | A4416 | Merck | 1:3000 - 1:6000 | |

**Supplemental Table S2.** Sequences and positions of two single guide RNAs (sgRNAs) used in CRISPR/Cas9 system.

| Orientation | sgRNA Cut Position (1-based) | sgRNA Sequence | sgRNA Context Sequence | PAM (Protospacer adjacent motif) Sequence |
|---|---|---|---|---|
| sense | 73 | CGAGCCAUGGCGGGGAACUG | CAGCCGAGCCATGGCGGGGAACTG**TGG**GGC | TGG |
| sense | 184 | GACGGCGCGCUGGGCUGGCG | CTGCGACGGCGCGCTGGGCTGGCG**CGG**CCT | CGG |

**Supplemental Table S3.** Primers used to develop and check the knockdown status of IRAK3 in THP-1 cells.

| Forward primers | | Reverse primers | | PCR product (bp) | |
|---|---|---|---|---|---|
| **Name** | **Sequence (5'-3')** | **Name** | **Sequence (5'-3')** | **Wild type** | **Knockout** |
| 41644-IRAK3_Fwd1 | GTGTTCCTAGGGCTCTGCTG | 41820-IRAK3_Rev1 | TACGGTTTGCAATGAACGCG | 737 | 625 |
| 41819-IRAK3_Fwd2 | CACACGCTGCTGTTCGAC | 41820-IRAK3_Rev1 | TACGGTTTGCAATGAACGCG | 579 | No amplification |
| 41644-IRAK3_Fwd1 | GTGTTCCTAGGGCTCTGCTG | 41645-IRAK3_Rev2 | GGGTGATTTTGCAAGGACCA | PCR products (601 bp) were cloned to TOPO vectors for DNA sequencing. | |
| T3 primer | ATTAACCCTCACTAAAGGGA | Used for DNA sequencing of TOPO vectors inserted with the checked sequences of IRAK3 gene from THP-1 wild type and knockdown cells. | | | |

**Supplemental Table S4.** PCR conditions.

| Step | PCR temperature (°C) | Time | Number of Cycles |
|---|---|---|---|
| Initial denaturation | 95 | 5 minutes | 1 |
| Denaturation | 95 | 30 seconds | 40 |
| Annealing | 56 | 30 seconds | |
| Extension | 72 | 30 seconds | |
| Final extension | 72 | 5 minutes | 1 |

**Supplementary Table S5**. Primers used in mutagenesis of IRAK3 constructs for complementation study.

| Mutagenesis primers | Primer sequence (5' to 3') |
|---|---|
| IRAK3R97A forward | 5'-CCTCCAGGAGATGGGACATCGTGCAGCTATTCATTTAATT-3' |
| IRAK3R97A antisense | 5'-AATTAAATGAATAGCTGCACGATGTCCCATCTCCTGGAGG-3' |
| IRAK3W74A forward | 5'-GTAAAAGTGGAACAAGAGAATTACTTGCGTCCTGGGCACAGA-3' |
| IRAK3W74A antisense | 5'-TCTGTGCCCAGGACGCAAGTAATTCTCTTGTTCCACTTTTAC-3' |

*Blue underlined nucleotides are mutations. The information for the primer used in Gateway cloning, sequencing and mutagenesis for mutants IRAK3 R372L can be found in (4).*

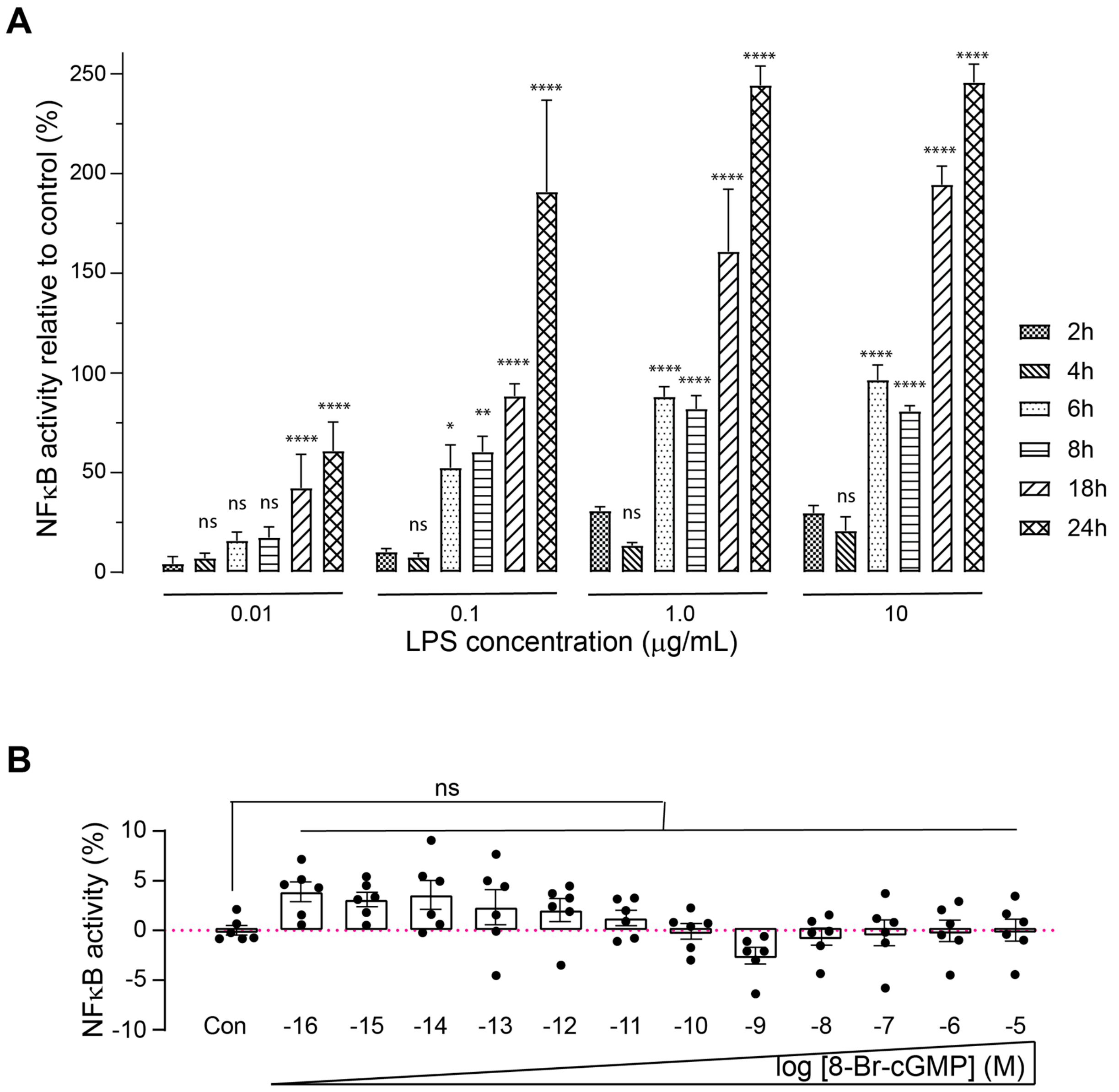


**Supplemental Figure S1.** NFκB activity in THP-1-BLUE cells after treatment with lipopolysaccharide (LPS) or 8-Br-cGMP. (A) NFκB activity in THP-1-BLUE cells was measured by induction of the NFκB-inducible SEAP reporter gene post-treatment with LPS at different time points (2h, 4h, 6h, 8h, 18h and 24h). LPS induced-NFκB activity changes were normalised to mock-induced cells (control). Data shown as mean ± SD (n = 6, the statistic analyses performed for each set of LPS concentrations and shown for comparisons between 2h point and other time points, One-way ANOVA followed by Tukey's multiple comparisons, *P<0.05, **P<0.01, ****P<0.0001, ns: not significant). (B) 8-Br-cGMP has no effect on NFκB activity in THP-1-BLUE cells that have not been treated with LPS. THP-1-BLUE cells were treated with 8-Br-cGMP at indicated concentrations for 24h, and NFκB activity was measured by induction of the NFκB-inducible SEAP reporter gene and normalised to cells not treated with 8-Br-cGMP (control, Con). Data shown as mean ± SEM (n = 6, One-way ANOVA followed by Dunnett's multiple comparisons test, ns: not significant).

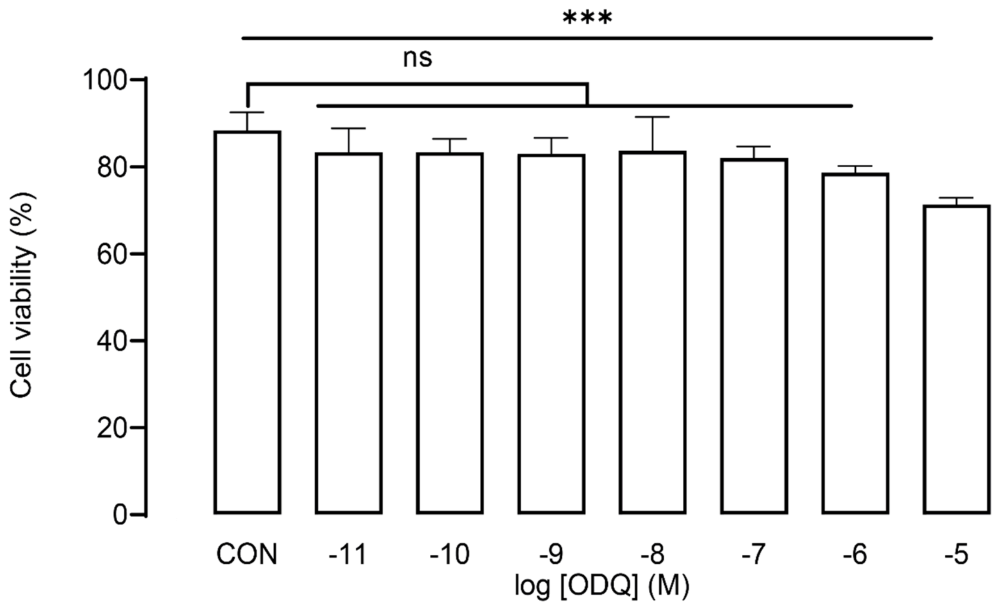


**Supplemental Figure S2.** THP-1 cell viability after ODQ treatment. THP-1 cells were treated with ODQ at indicated concentrations or treated with PBS as a control (CON) for 24h. After the treatment, cell viability was measured by staining with Trypan Blue and counting viable cells using the Countess II Automated cell counter (mean ± SD, n = 3, One-way ANOVA followed by Dunnett's multiple comparisons test, ***P<0.001, ns: not significant).

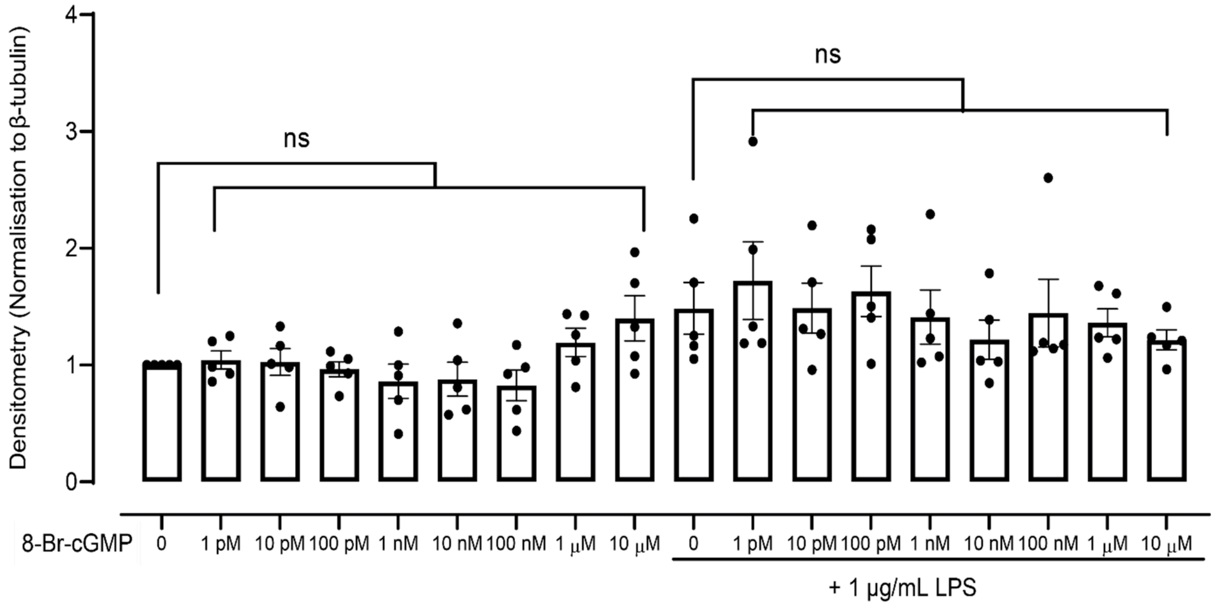


**Supplemental Figure S3.** IRAK3 relative protein expression in THP-1 cells after treatment with 8-Br-cGMP with or without LPS. THP-1 cells were treated with 8-Br-cGMP at indicated concentrations, in the presence or absence of 1 μg/mL LPS for 16-18h. After treatment, protein cell lysate were used for immunoblot analysis. Densitometry analysis of IRAK3 protein expression relative to β-tubulin level on immunoblots blots (mean ± SEM, n = 5, One-way ANOVA followed by Dunnett's multiple comparisons test, ns: not significant). Full length immunoblots used in this analysis are shown in Supplemental Figure S4.

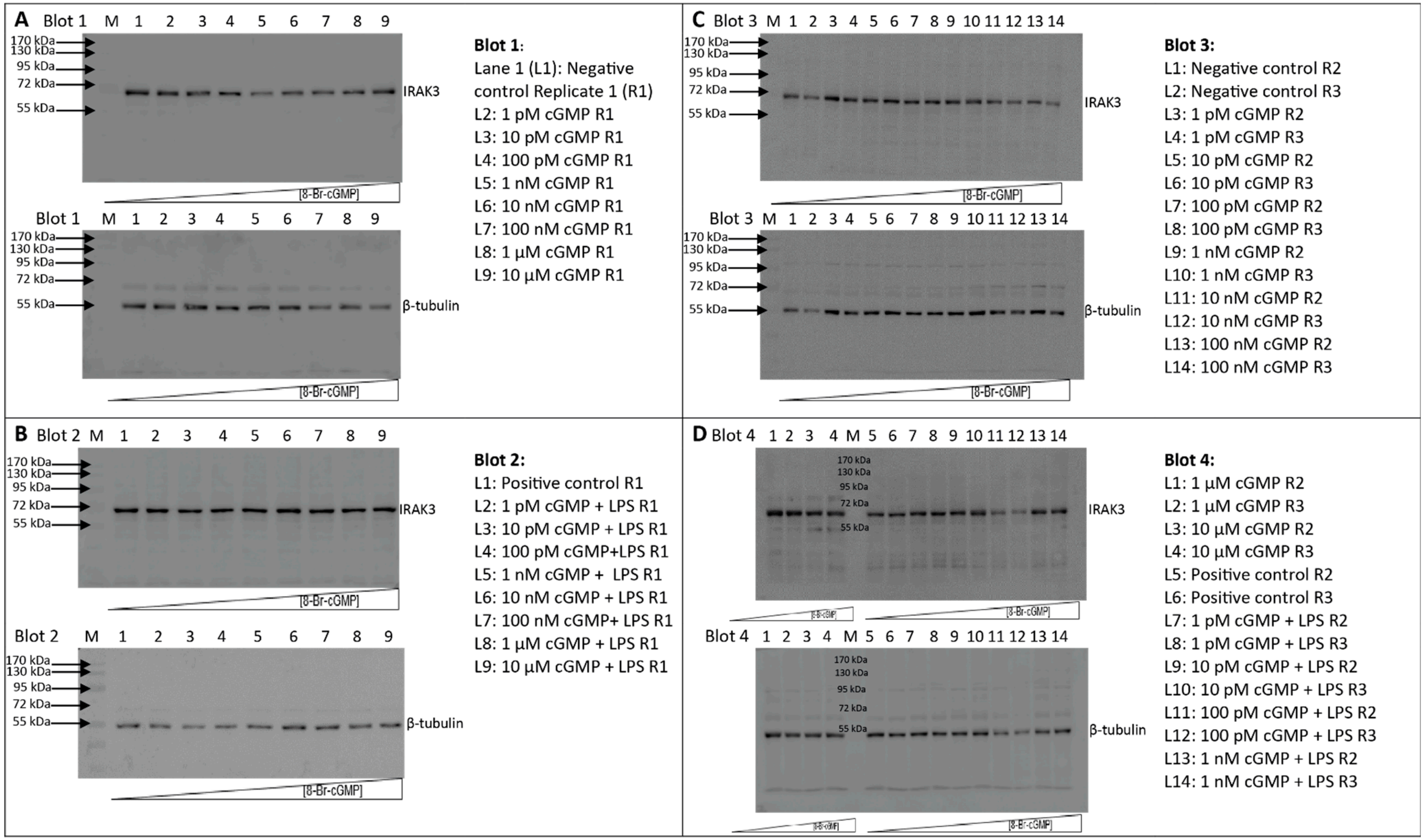


**Supplemental Figure S4 A-D.** Full length immunoblots of THP-1 cells after treatment with 8-Br-cGMP at indicated concentrations with or without LPS. The cells were stimulated with 1 µg/mL LPS or treated with PBS, and 16-18h post-treatment cell lysates were collected for Western blots to investigate IRAK3 protein expression. The blots were incubated with primary IRAK3 antibodies from Cell Signaling Technology. Densitrometric analysis of this data is reported in Supplemental Figure S3. ***Parts E-H continue***.

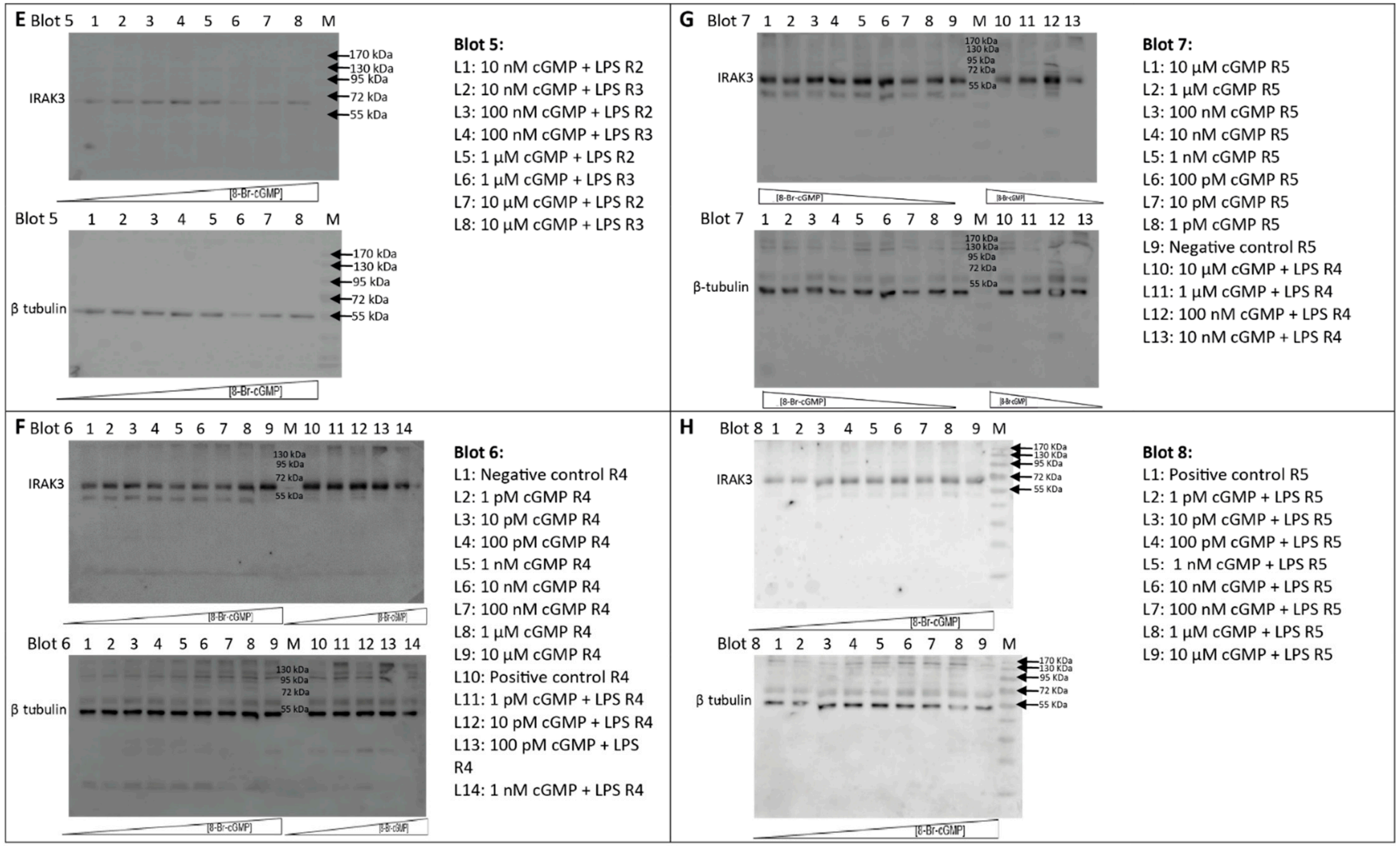


**Supplemental Figure S4.** (cont.) Full length immunoblots of THP-1 cells after treatment with 8-Br-cGMP at indicated concentrations with or without LPS. The cells were stimulated with 1 μg/mL LPS or treated with PBS, and 16-18h post-treatment cell lysates were collected for Western blots to investigate IRAK3 protein expression. The blots were incubated with primary IRAK3 antibodies from Cell Signaling Technology. Densitrometric analysis of this data is reported in Supplemental Figure S3.

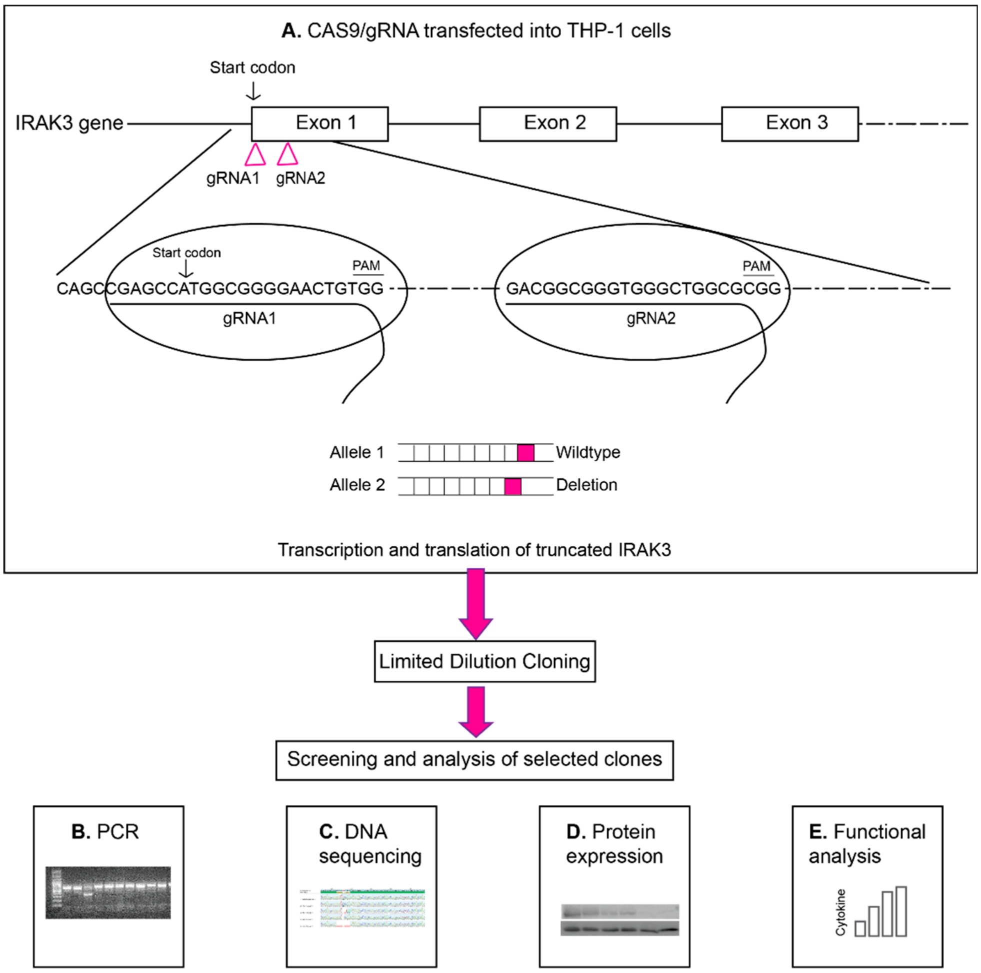


**Supplemental Figure S5**. CRISPR/Cas9 system used to knock down IRAK3 gene. (A) The mixture of two sgRNAs and Cas9-GFP protein were transfected to THP-1 cells. The Cas9-sgRNA(s) complex binds to genomic DNA and cuts at the target sequences as shown in panel A; this leads to deletion of nucleotide(s) causing frameshift mutation(s). The frameshift mutation(s) can result in early stop-codon, leading to transcription and translation of truncated IRAK3 protein lacking most domains. The Cas9-sgRNAs transfected THP-1 cells underwent limited dilution cloning to generate IRAK3 knockdown cell lines. To verify the knockdown efficacy, the potential IRAK3 knockdown cell lines were analysed by PCR (B), DNA sequencing (C), protein expression using immunoblots (D) and functional analysis using ELISA to measure cytokine production (E).

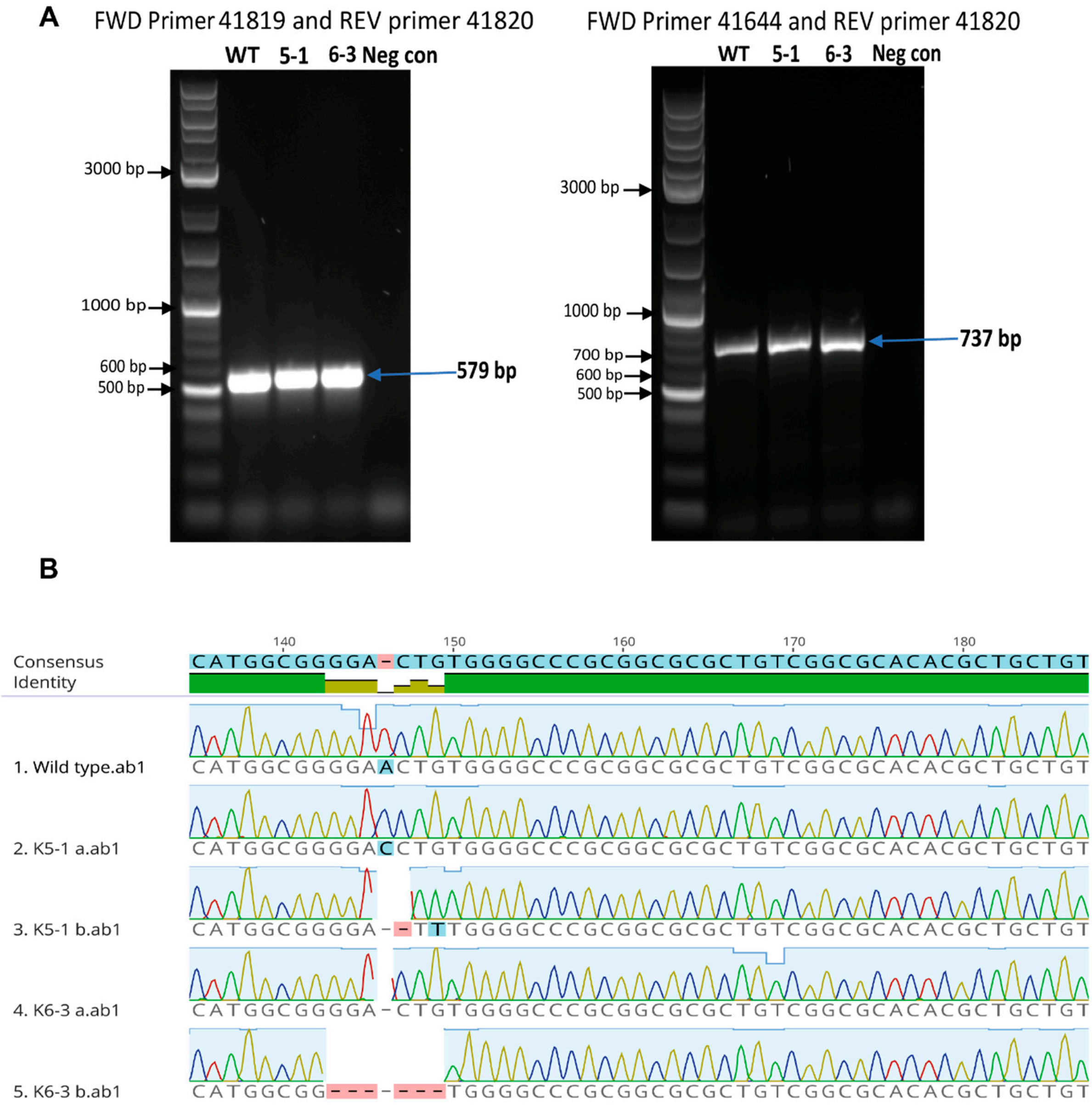


**Supplemental Figure S6**. Screening and analysis of the knockdown of IRAK3 gene. (A) PCR for checking the efficiency of gDNA targeting of two gRNAs. Genomic DNA was extracted and used for PCR with two sets of primers 41819-IRAK3_Fwd2/41820-IRAK3_Rev1 and 41644-IRAK3_Fwd1/41820-IRAK3_Rev1 as listed in Supplemental Table 3. PCR analysis shows the cleavage occurred only at one of two sites for sgRNAs. (B) DNA sequences of IRAK3 gene of THP-1 wild type, K5-1 and K6-3 cell lines. Genomic DNA were extracted from cells and used for a PCR run with primers 41644-IRAK3_Fwd1/41645-IRAK3_Rev2 listed in Supplemental table 3. PCR products were cloned into TOPO vectors and the vectors with inserts were used for DNA sequencing. K5-1 is a heterozygous mutant with a substitution of one nucleotide at an allele and a deletion of two nucleotides at the other allele. K6-3 is a heterozygous mutant with a deletion of one nucleotide at an allele and a deletion of seven nucleotides at the other allele.

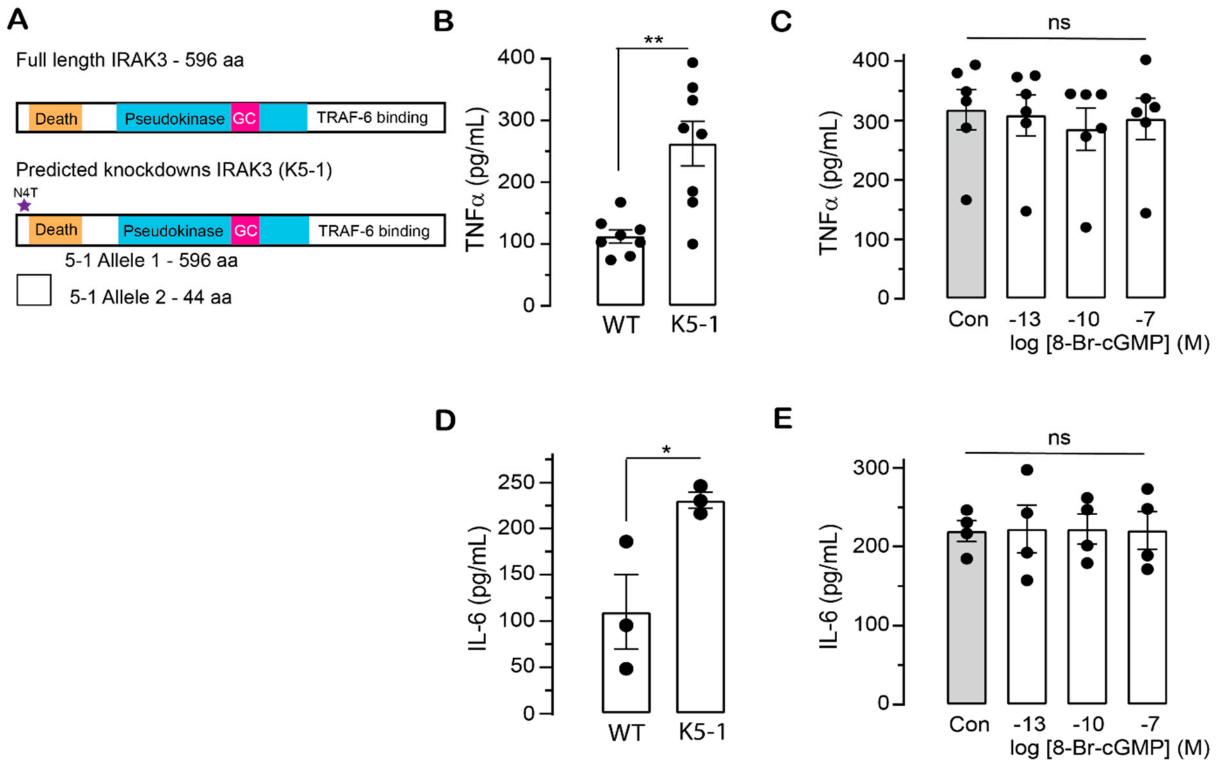


**Supplemental Figure S7**. Effect of cGMP on inflammatory responses in CRISPR mediated-IRAK3 knockdown cell line K5-1. (A) The predicted knockdowns of IRAK3 in K5-1 cell line contain one allele with 596 amino acids with one amino acid substitution (N4T) containing the wild type protein domains, and a second allele containing a unique set of 44 amino acids. (B) and (D) LPS-induced TNF-α and IL-6 levels were significantly increased in K5-1 THP-1 IRAK3$^{+/-}$ cells compared to THP-1 IRAK3$^{+/+}$ wild type cells. THP-1 IRAK3$^{+/+}$ wild type cells and K5-1 IRAK3$^{+/-}$ cells were stimulated with 1 μg/mL LPS for 18h. After treatment cell supernatants were collected for quantification of TNF-α (B) and IL-6 (D) cytokine production (mean ± SEM, n = 3-8, Unpaired t-test, *P<0.05, **P<0.01). (C) and (E) cGMP has no effect on cytokine production K5-1 THP-1 IRAK3$^{+/-}$ cells. K5-1 THP-1 IRAK3$^{+/-}$ cells were treated with 1 μg/mL LPS and 8-Br-cGMP at 100 fM ($10^{-13}$ M), 100 pM ($10^{-10}$ M) and 100 nM ($10^{-7}$ M), after 24h treatment cell supernatants were collected for quantification of TNF-α (C) and IL-6 (E) protein production using ELISA, control cells treated with LPS only (mean ± SEM, n = 4-6, One-way ANOVA followed by Dunnett's multiple comparisons test, ns: not significant).

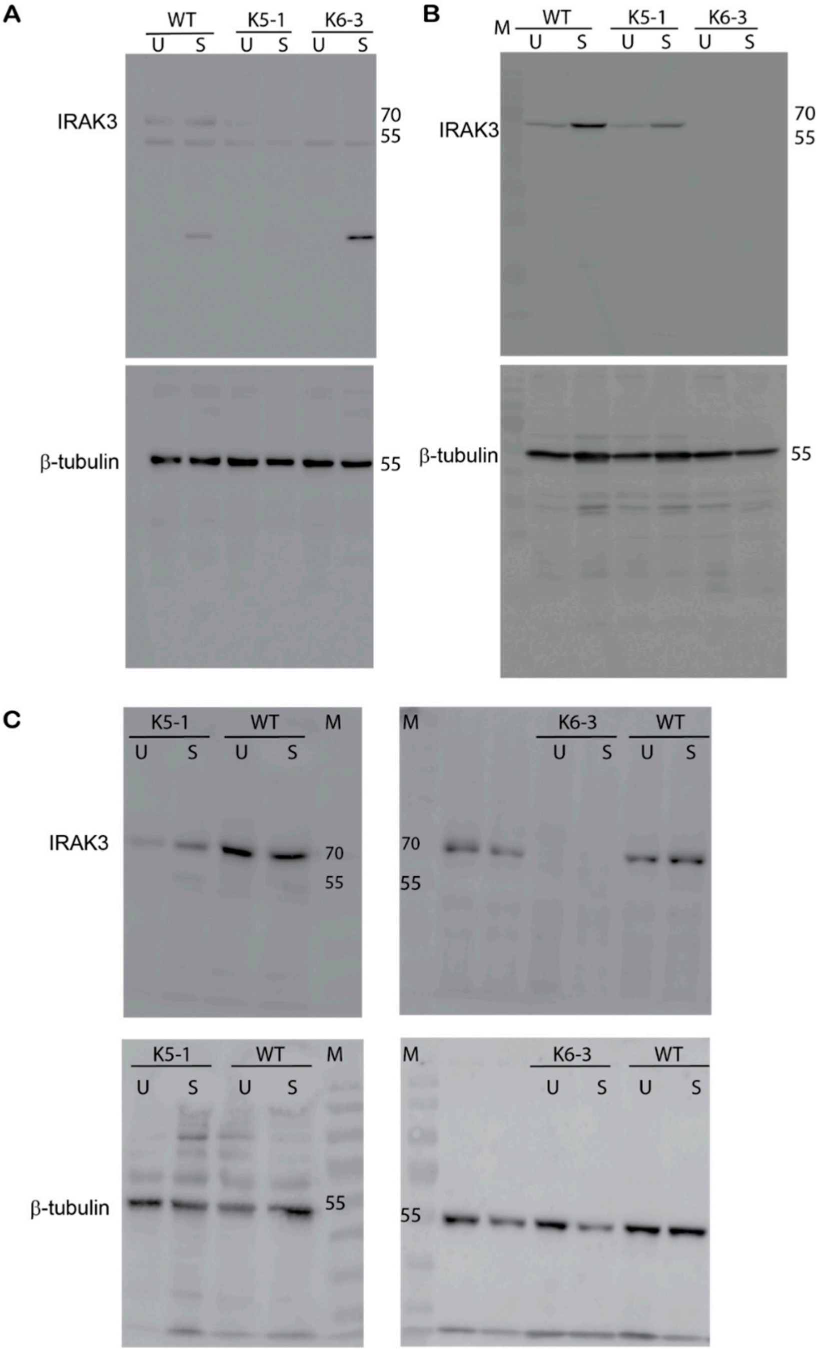


**Supplemental Figure S8.** Full length immunoblots of THP-1 IRAK3$^{+/+}$ cells (wild type, WT) and IRAK3$^{+/-}$ (K5-1) or IRAK3$^{-/-}$ (K6-3) cell lines. The cell lines were stimulated with 1 μg/mL LPS (S) or treated with PBS (unstimulated – U), 16-18h post-treatment cell lysates were collected for Western blots to investigate IRAK3 protein expression. The blots were incubated with primary IRAK3 antibodies from three commercial brands including NSJ Bioreagent (A), CST (B), and Santa Cruz (C).

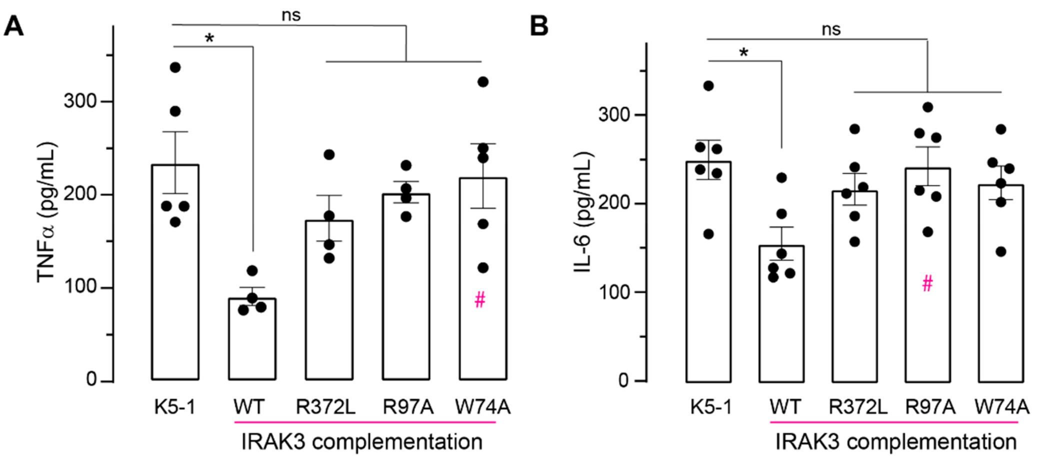


**Supplemental Figure S9**. Effect of mutations in guanylate cyclase centre (R372L) and death domain (W74A, R97A) of IRAK3 on cytokine production. K5-1 THP-1 IRAK3$^{+/-}$ cells were transfected with vectors carrying gene encoding wild type (WT) or mutated versions of IRAK3; untransfected K5-1 THP-1 IRAK3$^{+/-}$ were used as a negative control. After transfection, the transfected and negative control cells were treated with 1 µg/mL LPS for 24h, and cell supernatants were collected for quantification of TNF-α (A) and IL-6 (B) protein levels using ELISA (mean ± SEM, n = 4-6, One-way ANOVA followed by Tukey's multiple comparisons test; *P<0.05, ns: not significant, pink hash mark (#) indicates the statistically significant difference between WT and other IRAK3 mutants, $^{\#}$P<0.05).

## References

1. Geng D, Ciavattone N, Lasola JJ, Shrestha R, Sanchez A, Guo J, et al. 2020. Induction of IRAK-M in melanoma induces caspase-3 dependent apoptosis by reducing TRAF6 and calpastatin levels. *Commun. Biol*. 3(1):306.
2. Miyata M, Lee J-Y, Susuki-Miyata S, Wang WY, Xu H, Kai H, et al. 2015. Glucocorticoids suppress inflammation via the upregulation of negative regulator IRAK-M. *Nat. Commun.* 6:6062.
3. Zhang K, Zhang Y, Gu L, Lan M, Liu C, Wang M, et al. 2018. Islr regulates canonical Wnt signaling-mediated skeletal muscle regeneration by stabilizing Dishevelled-2 and preventing autophagy. *Nat. Commun.* 9(1):5129.
4. Freihat L, Wheeler J, Wong A, Turek I, Manallack D, Irving H. 2019. IRAK3 modulates downstream innate immune signalling through its guanylate cyclase activity. *Sci. Rep.* 9(1):15468.
